# microRNA Profiling of Amniotic Fluid: Evidence of Synergy of microRNAs in Fetal Development

**DOI:** 10.1371/journal.pone.0153950

**Published:** 2016-05-11

**Authors:** Tingting Sun, Weiyun Li, Tianpeng Li, Shucai Ling

**Affiliations:** 1 Institute of Neuroscience and Anatomy, Zhejiang University School of Medicine, Hangzhou, 310058, China; 2 College of Environmental Science and Engineering, Donghua University, Shanghai, 201620, China; Ecole Normale Superieure de Lyon, FRANCE

## Abstract

Amniotic fluid (AF) continuously exchanges molecules with the fetus, playing critical roles in fetal development especially *via* its complex components. Among these components, microRNAs are thought to be transferred between cells loaded in microvesicles. However, the functions of AF microRNAs remain unknown. To date, few studies have examined microRNAs in amniotic fluid. In this study, we employed miRCURY Locked Nucleotide Acid arrays to profile the dynamic expression of microRNAs in AF from mice on embryonic days E13, E15, and E17. At these times, 233 microRNAs were differentially expressed (*p*< 0.01), accounting for 23% of the total *Mus musculus* microRNAs. These differentially-expressed microRNAs were divided into two distinct groups based on their expression patterns. Gene ontology analysis showed that the intersectional target genes of these differentially-expressed microRNAs were mainly distributed in synapse, synaptosome, cell projection, and cytoskeleton. Pathway analysis revealed that the target genes of the two groups of microRNAs were synergistically enriched in axon guidance, focal adhesion, and MAPK signaling pathways. MicroRNA-mRNA network analysis and gene- mapping showed that these microRNAs synergistically regulated cell motility, cell proliferation and differentiation, and especially the axon guidance process. Cancer pathways associated with growth and proliferation were also enriched in AF. Taken together, the results of this study are the first to show the functions of microRNAs in AF during fetal development, providing novel insights into interpreting the roles of AF microRNAs in fetal development.

## Introduction

AF is the fluid in the amniotic sac that appears 7–8 days after fertilization [1]. So, until delivery, the growth and development of the fetus occurs in its presence. It is widely accepted that AF provides movement space and mechanical protection for the fetus [2]. However, a large body of evidence suggests that its components play more important roles in fetal development [3, 4]. The composition of AF reflects the dynamic status of the developing fetus, and specific components serve as indicators of specific conditions in fetal development as well as malformations of the nervous system [5, 3, 6].

AF is in direct contact with the fetal skin. In terms of molecular interchange efficiency and the immunological barrier, signaling molecules are likely to pass from the AF to act on the fetus [7, 8], especially through the non-keratinized fetal skin [9]. Among its components, RNA has received much attention since genome-wide methods have been used to analyze their dynamic changes in AF. Genomic analysis of cell-free fetal RNA from AF offers much important information on fetal development, physiology, and pathology during pregnancy [10–12]. Notably, a transcriptome study showed that neurodevelopment-related genes are abundant in human mid-trimester AF, reflecting the active stages of neuronal development at this time [13]. Furthermore, the “-omics” strategy has been used to study the dynamic changes of RNAs and proteins in AF, providing novel biomarkers and therapeutic targets for prenatal diagnosis and *in utero* treatment [14, 5].

MicroRNAs, a class of 22- to 24- nt non-coding RNAs, are transcriptional and post-transcriptional regulators that play critical roles in organ development and the formation of the nervous system [15–17]. It is notable that exogenous microRNAs loaded in microvesicles can be transferred to target cells [18, 19]. Based on these findings, we hypothesized that microRNAs in AF act as modulators to regulate the expression of specific genes during fetal development. To explore the functions of AF microRNAs in fetal development, we profiled their dynamic expression in mice on embryonic days E13, E15, and E17. Then we investigated their global roles by careful bioinformatics analysis. Finally, we validated the expression of representative microRNAs and their targets in the most enriched pathway, axon guidance, by quantitative real-time PCR.

## Materials and Methods

### Animals

Eight-week-old male and female C57BL/6 mice were obtained from the Shanghai Laboratory Animal Center (Chinese Academy of Science, Shanghai, China). Animal care and use were in accordance with the guidelines of the China Committee on Animal Experiments and were approved by the Zhejiang University Animal Care and Use committee (Approval number ZJU2015-428-01). All mice were housed in a central facility and maintained under controlled humidity and temperature, with standard alternating 12-h periods of light and darkness. Animals had free access to water and food. After three days of acclimatization, pairs of females were kept with single males for 12 h overnight. On the morning of the test day, the simultaneous presence of a vaginal plug and spermatozoa in the vaginal cytology were taken to indicate day 0 of gestation. Pregnancy was further confirmed by weight gain. All efforts were made to minimize the suffering of animals and the number of animal used.

### Sample Preparation and Microarray Arrays

On days 13, 15, and 17 of gestation, 6 animals randomly selected from each group were deeply anesthetized with pentobarbital sodium (45mg/kg, cas 57-33-0, Merck, Darmstadt, Germany) and then AF was carefully collected in a 1-ml sterilized syringe after abdominal exposure with minimal trauma. Total RNA was harvested using TRIzol (Invitrogen, Carlsbad, CA) and an RNeasy mini kit (Qiagen, Hilden, Germany) according to the manufacturers’ instructions. Before labelling with the miRCURY Hy3/Hy5 Power labelling kit, the yield and integrity of total RNA were assessed using a spectrometer (ND-2000, NanoDrop Technologies Inc., Rockland, DE) and gel electrophoresis. Then the samples were hybridized on miRCURY LNA arrays (ver.16.0; Exiqon, Vedbaek, Denmark) and after washing steps, the slides were imaged on a GenePix 4000B microarray scanner and raw data were acquired from the scanned images using GenePix Pro 6.0 software (Axon Instruments, Foster City, CA, USA). We have submitted the microarray data to the Gene Expression Omnibus (Accession No. GSE70324)

### Hierarchical Clustering and Expression Patterns Analysis

Raw data were normalized using median normalization and the differentially-expressed microRNAs were assessed using one-way analysis of variance (ANOVA) followed by the Bonferroni multiple-comparisons test. Values of *p* <0.01 were considered to be statistically significant. Hierarchical clustering was performed using the average-linkage algorithm in Mev software (ver.4, TIGR). The expression patterns were determined using the self-organizing map algorithm, which computes the Euclidean distance from a specific input node to each of the other nodes using nearest-neighbor rules.

### Target Prediction

Target prediction for the two categories of microRNAs was performed using the algorithms Targetscan, Pita, and miRanda. Detailed information on these algorithms is available in a previous report [20]. In brief, Targetscan and miRanda predictions are based on the matching of sites between the seed regions of microRNAs and mRNAs, while Pita prediction is based on target-site accessibility. All the prediction processes were conducted using custom-written executable files that computed the parameters between microRNAs and mRNAs based on the inherent algorithms and the set thresholds. The thresholds for the algorithms were: scores ≥70 for Targetscan; ∆∆G ≤-5 for Pita; and seed = 7 for miRanda. The intersections of the output results of the three algorithms were used as prediction results for the differentially-expressed microRNAs.

### Gene Annotation and Enrichment Analysis

The annotation and enrichment analyses of target genes were performed using DAVID web server tools (https://david.ncifcrf.gov/). The targets of the two groups of microRNAs were separately submitted to DAVID for annotation and enrichment analyses. The main components of annotation in Gene Ontology (GO) mainly provided the cellular locations and biological functions of validated microRNA targets. The GO-biological processes and GO-cellular component analyses were performed using Fisher’s exact test and the χ^2^ test, where both the Expression Analysis Systematic Explorer and the False Discovery Ratio (FDR) were calculated to correct the *p* value. The enrichment was calculated as previously described [20] and the whole mouse genome was used as the background. Only terms with both a *p* value and an FDR≤ 0.01 were considered to be significant.

### Pathway Analysis

Pathway analysis was based on the Kyoto Encyclopedia of Genes and Genomes (KEGG) database; similarly, we used Fisher’s exact test and the χ^2^ test to identify significant pathways, and terms with both a *p* value and an FDR ≤0.01were considered to be significant. Enrichment was calculated as for the GO terms above. To further analyze the interactions between networks, we used Cytoscape software (ver.2.8.0, Cytoscape consortium, USA) and the network advanced network merge plug-in (ver.3.0) to analyze the interactive microRNAs and mRNAs between networks. The top enriched pathway was analyzed using the integrated network analysis plug-in, which calculated the basic network parameters of the pathways. The parameter of betweenness centrality, which reflects the “weight” of one node in the network, was used as a scale of node size. Another parameter, closeness centrality, which reflects the closeness between nodes, was used as a color scale for node gradients.

### Quantitative Real-Time PCR

On days 13, 15, and 17 of gestation, 4 animals randomly selected from each group were deeply anesthetized with pentobarbital sodium and then the collection of AF and the extraction of total RNA was performed in the same way as described in microarray part. The expression of representative microRNAs and their predicted targets in axon guidance pathway were determined by two steps of quantitative RT-PCR. First, the cDNA of microRNAs was synthesized with specific reverse-transcriptional primers (S1 Table) and the cDNAs of mRNAs were synthesized with oligo(dT). Then PCR reactions containing SYBR Green Mix (Invitrogen, Carlsbad, CA) were performed on a CFX96 system (Bio-Rad, Hercules, CA) with specific amplification primers (S2 Table). The relative microRNA and mRNA levels were computed using the 2^-ΔΔCt^ method, where Snord2 and NADPH were used as internal controls for microRNA and mRNA, respectively. All reactions were run in triplicate.

### Statistical Analysis

All quantitative data are presented as mean±SD and differences were assessed using one-way ANOVA followed by the Bonferroni multiple-comparison test. Values of *p*<0.01were considered to be statistically significant and all analyses were performed using SPSS (ver.20.0, SPSS Inc., Chicago, IL).

## Results

### Two Distinct Expression Patterns of microRNAs on E13, E15, and E17

**Fig 1:**
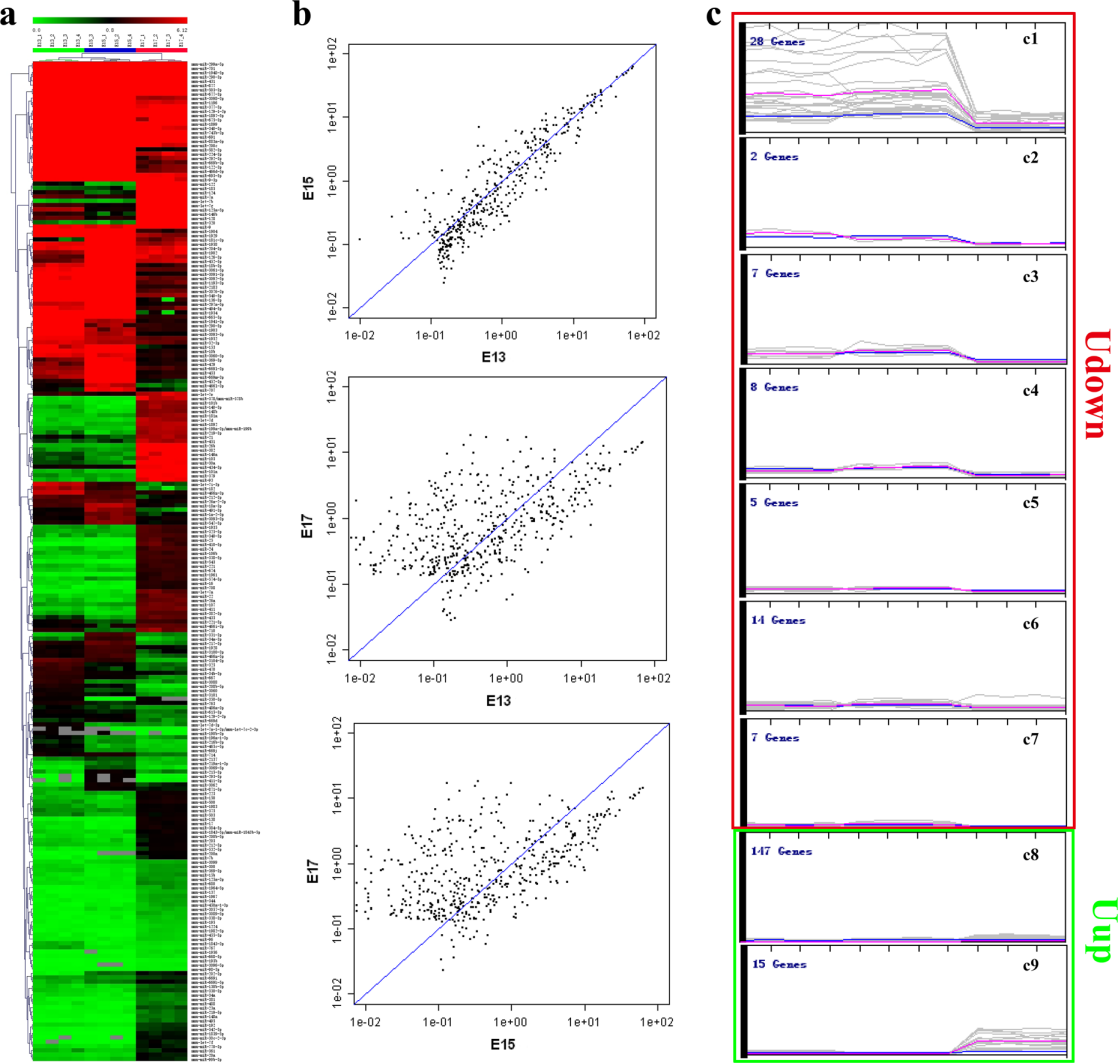
MicroRNA expression pattern analyses. (a). Heat-map and hierarchical clustering of differentially-expressed microRNAs in amniotic fluid on E13, E15, and E17, tested by one-way ANOVA, *p*<0.01.(b) Correlation scatter-plots of microRNA expression on E13, E15, and E17. (c) Expression patterns of microRNAs analyzed using self-organizing map algorithm. The Udown group is contained seven clusters (c1-c7) and the Uup microRNA group contained two clusters (c8 and c9).

MicroRNA expression at E13, E15, and E17 was investigated using the large-scale and highly-specific miRCURY LNA arrays. Signals from arrays at the three time-points were subjected to ANOVA analysis after normalization and this showed that 233 microRNAs were differentially expressed (*p* < 0.01) at E13, E15, and E17, accounting for 23% of the total *Mus musculus* microRNAs. Hierarchical clustering showed good consistency within the same group and revealed distinct expression clusters with time (Fig 1A). Based on the correlation plots at the 3 time points, the levels of microRNAs on E17 were distinct from those on E13 and E15 (Fig 1B). Further, these differentially-expressed microRNAs were divided into 9 clusters based on their expression patterns by the self-organizing map algorithm. As clusters c1 to c7 had a similar expression tendency from E13 to E17, they were merged into one group defined as ‘Udown’. Clusters c8 and c9 were merged into another group and defined as‘Uup’ according to their similar expression tendency (Fig 1C). Thus, the differentially-expressed microRNAs were divided into two distinct groups. Uup was characterized by dramatically increased expression on E17 and this group included 162 microRNAs, while Udown had a corresponding reversed expressional pattern and was composed of 71 microRNAs (Fig 1C).

### Targets of the Two Groups of microRNAs

**Fig 2:**
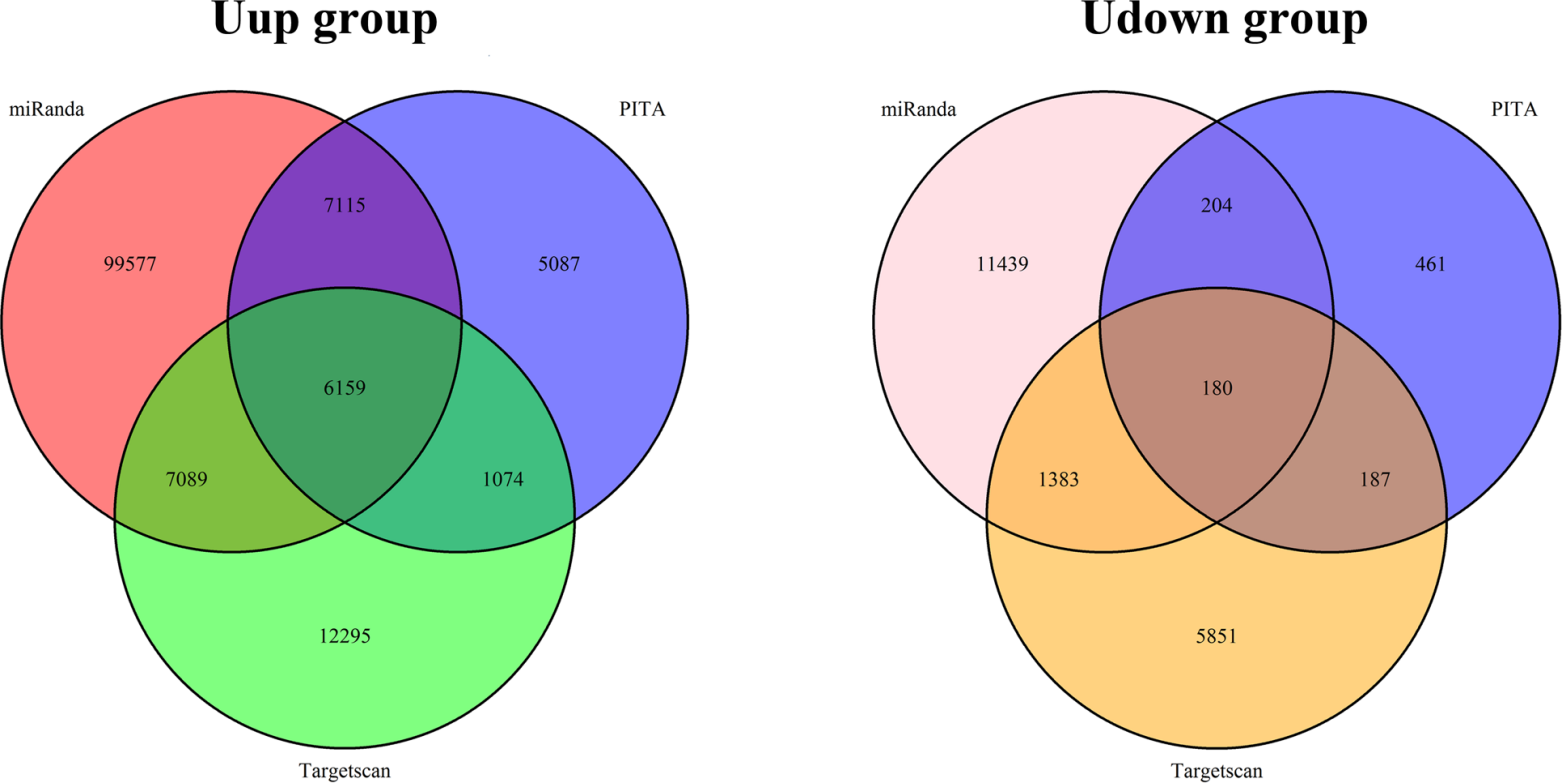
Targets of the differentially-expressed microRNAs. Venn diagrams of the targets of the microRNAs in the Uup and Udown groups. Target prediction was performed using Targetscan, Pita, and miRanda. The thresholds were scores ≥70 for Targetscan, ∆∆G ≤-5 for Pita, and seed = 7 for miRanda.

To understand the functions of these differentially-expressed microRNAs, the targets of the differentially-expressed microRNAs in the two groups were calculated using the target-prediction algorithms. In order to enhance the specificity and reliability of prediction, we combined the advantages of the algorithms Targetscan, miRanda, and Pita and used the intersections for further analysis. There were 6159 unique intersecting microRNA-mRNA pairs and 2609 final unique genes for the Uup microRNAs. There were fewer targets for the Udown microRNAs: 180 unique microRNA-mRNA pairs and 157 final unique genes (Fig 2), consistent with the reduced diversity of this group of microRNAs (S3 Table).

### Function and Pathway Analysis of Two Categories of microRNAs

**Fig 3:**
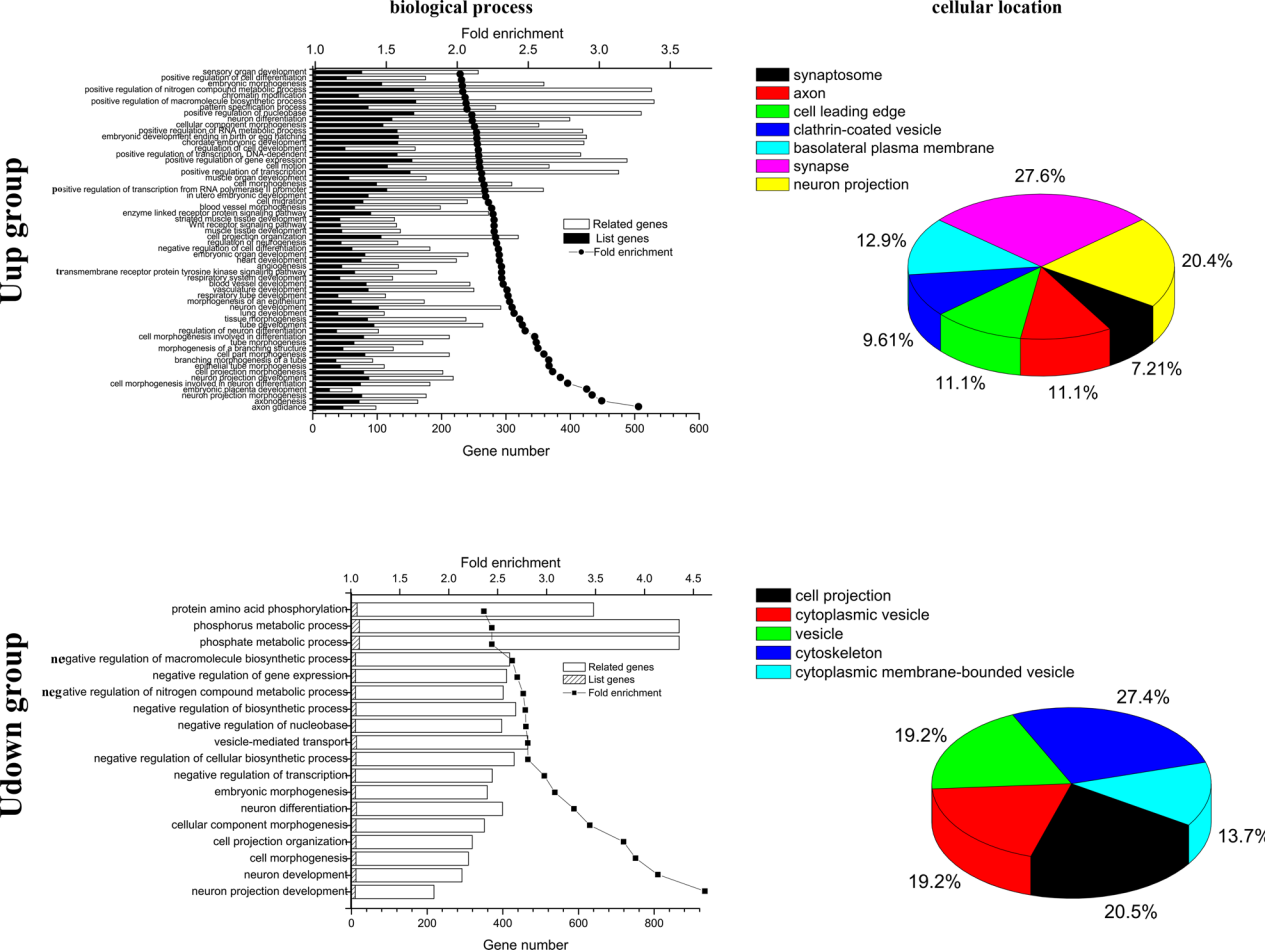
Biological functions and cellular location analysis of the targets of differentially-expressed microRNAs. Left upper panel: The top 58 terms of GO-Biological Process (BP) with fold enrichment >2, *p*<0.001, and FDR <0.001 in the Uup group. Left lower panel: the top 18 terms of GO-BP terms with fold enrichment >2 and *p*<0.01 in the Udown group. Upper *y*-axis, fold enrichment; lower *y*-axis, gene numbers for each term; black and hatched columns, numbers of genes in the list of targets; white columns, numbers of genes not in the list of target genes but associated with the corresponding term. Right panels: terms of GO-Cellular Component with fold enrichment >2, *p* <0.001, and FDR<0.01 in the Uup group (upper panel) and with fold enrichment >2, *p* <0.01 in the Udown group (lower panel).

To identify the functions of AF in development, the targets of the two categories were carefully analyzed using GO annotation and KEGG enrichment. These analyses were performed by mapping the predicted target genes from the differentially-expressed AF microRNAs to the GO and KEGG databases. The results showed that axon guidance was the most dominant term among 58 highly-enriched GO biological processes for Uup microRNAs (all *p* <0.001 and FDR <0.001, fold enrichment >2.0). The target genes of the Uup microRNAs were mainly distributed in synaptosome, axon, cell leading edge, clathrin-coated vesicle, basolateral plasma membrane, synapse, and neuron projection of cells (*p*<0.01 and FDR<0.01, fold enrichment >2.0), with the most -abundant locations for synapse and neuron projection that constituted 27.6% and 20.4% of the total genes, respectively. Consistently, neuron projection development was the most the highly-enriched term among the 17 highly-enriched GO biological processes for Udown microRNAs (*p* <0.001 and fold enrichment >2.0). The target genes of Udown microRNAs were mainly located in cell projection, cytoplasmic vesicle, vesicle, cytoskeleton, and cytoplasmic membrane-bounded vesicle of cells (*p*<0.01 and fold enrichment >2.0). Among these cellular components, cytoskeleton and cell projection of cells were the most concentrated locations, constituting 27.4% and 20.5% of the total genes, respectively (Fig 3).

**Fig 4:**
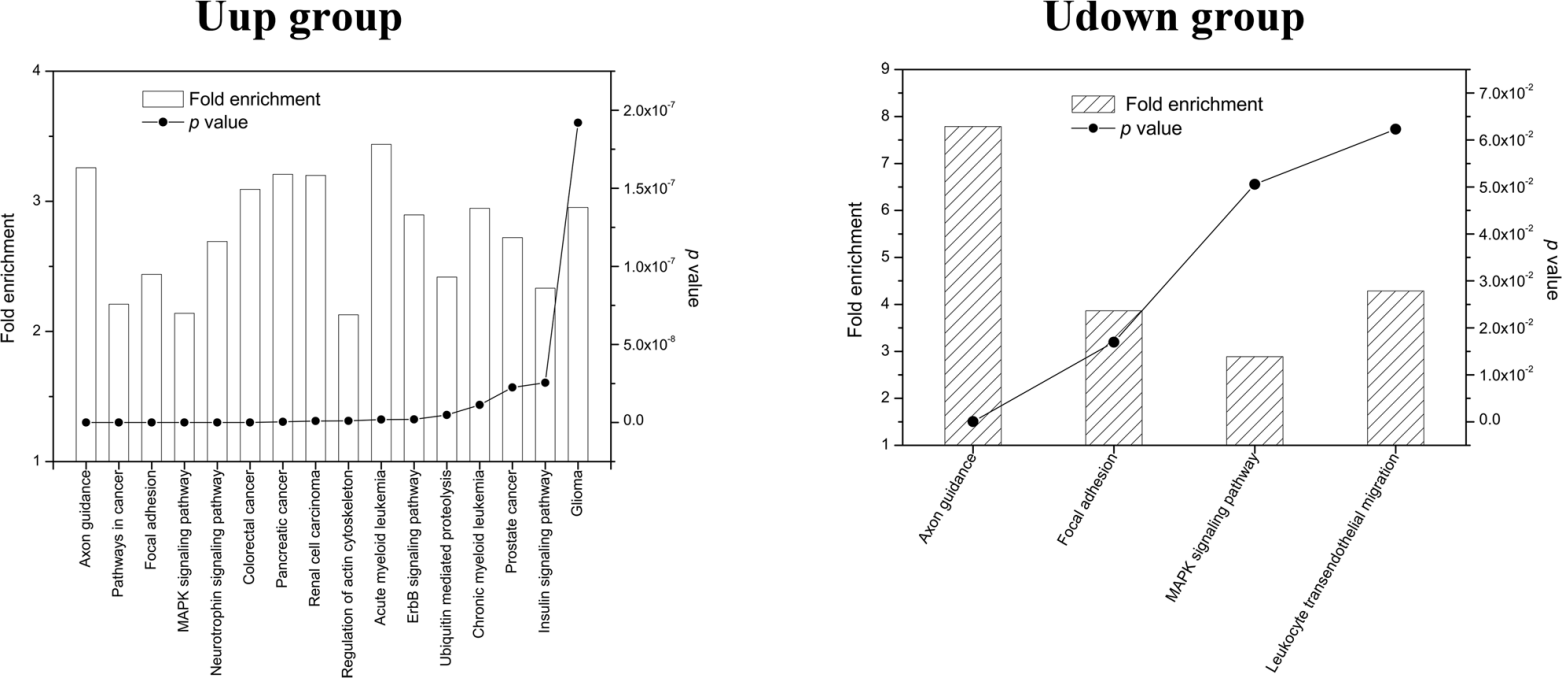
Pathway analysis of the targets of differentially-expressed microRNAs. Left panel: the top 16 terms of pathways with fold enrichment > 2, *p*<2×10^−7^, and FDR< 0.001 in the Uup group. Right panel: the top 4 terms of pathways with fold enrichment> 2 and *p*<0.07 in the Udown group. Left *y*-axis, fold enrichment; right *y*-axis, *p* value. Higher fold enrichment and lower *p* value indicate a greater degree of enrichment.

To analyze the functions of these differentially-expressed microRNAs, their interactional targets were explored in the KEGG database. The results showed that axon guidance was the most reliably enriched pathway for Uup microRNA targets (*p* = 1.17×10^−18^, FDR = 1.42 ×10^−15^, and fold enrichment = 3.26), consistent with the GO biological process results. Acute myeloid leukemia (*p* = 1.94×10^−9^, FDR = 2.36×10^−6^, and fold enrichment = 3.44), pancreatic cancer (*p* = 4.75×10^−10^, FDR = 5.76×10^−7^, and fold enrichment = 3.21), and renal cell carcinoma (*p* = 9.94×10^−10^, FDR = 1.21×10^−6^, and fold enrichment = 3.20) were relatively highly-enriched pathways. Notably, for Udown targets, axon guidance was also the most highly-enriched pathway (*p* = 5.22 ×10^−5^, FDR = 0.053, and fold enrichment = 7.79), consistent with the Uup results. Furthermore, the other pathways, such as focal adhesion and MAPK signaling, were also highly-enriched in both groups (Fig 4).

### Synergistic Effects of microRNAs in the Top Enriched Pathways

**Fig 5:**
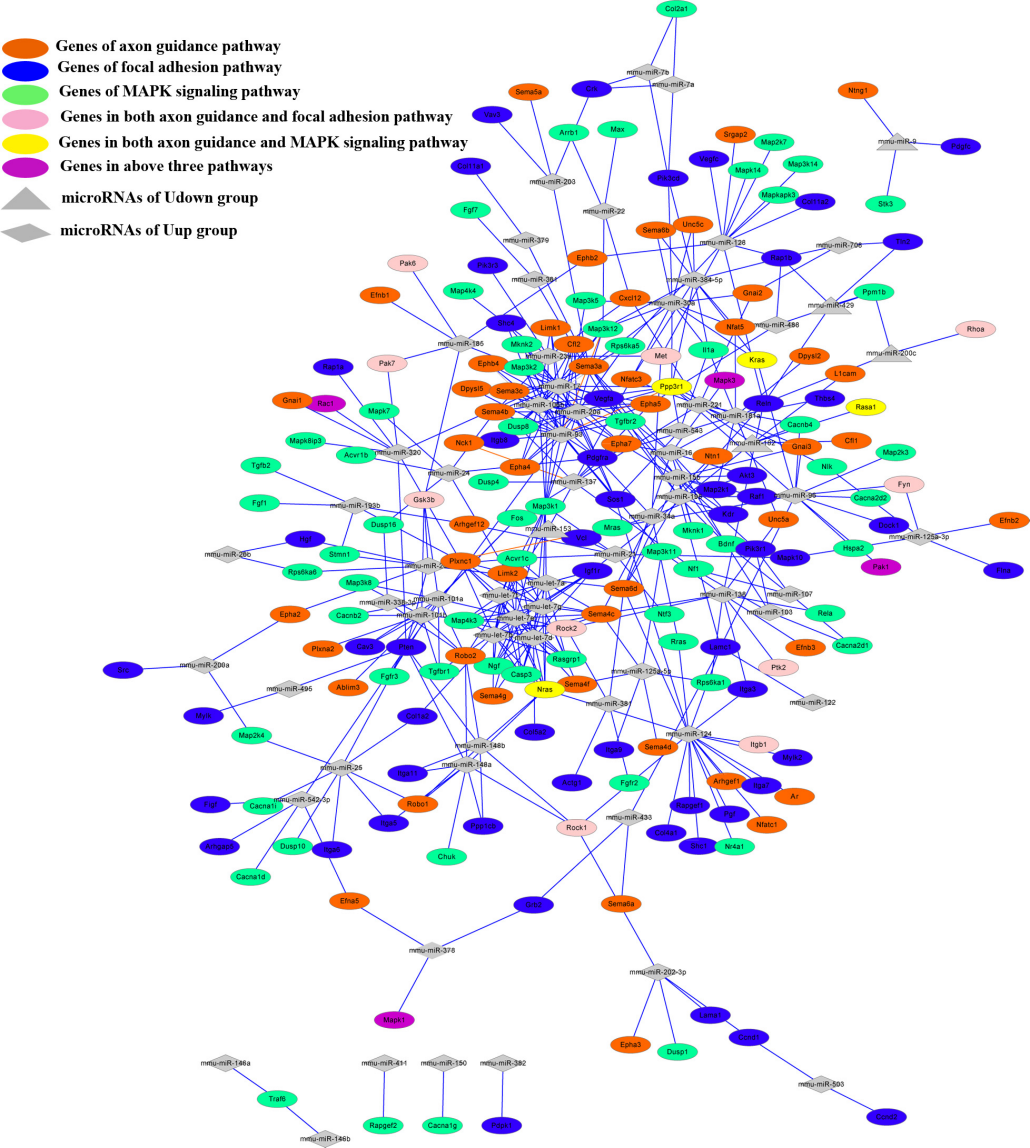
microRNA-mRNA interactions inthe axon guidance, focal adhesion, and MAPK signaling pathways. Merged microRNA-mRNA network of the three pathways. Red, blue, and green ellipses, target genes involved in axon guidance, focal adhesion, and MAPK signaling pathways, respectively. Pink ellipses, overlapped target genes in the axon guidance and focal adhesion pathways; yellow ellipses, overlapped target genes in the axon guidance and MAPK signaling pathways; violet ellipses, overlapped target genes in all three pathways; gray triangles, microRNAs of the Uup group; gray diamond, microRNAs of the Udown group.

Although the expression patterns of the two categories of microRNAs were inversely correlated, their overall functions were the same. To explore the interactions that led them to participate in the same functions, we transformed them and their targets into microRNA-mRNA regulatory networks with Cytoscape, using the internal algorithm Prefuse Force-Directed Layout. The merged network of the three networks for the axon guidance, focal adhesion, and MAPK signaling pathways revealed that they had close interactions, due to the common genes and microRNAs involved (Fig 5). According to KEGG enrichment, 14 genes simultaneously participated in the axon guidance and focal adhesion pathways. Among these genes, *Rhoa* was targeted by miR-200cin the Udown group and the other 13 genes were targeted by microRNAs in the Uup group. Eight genes simultaneously participated in the axon guidance and MAPK signaling pathways, among which *Rasa1*was targeted by miR-182 in the Udown group and the other 7 genes were targeted by microRNAs in the Uup group.*Rac1*, *Pak1*, *Mapk3*, and *Mapk1*targeted by microRNAs in the Uup group were predicted to be involved in all three of the above pathways (Table 1). Besides the genes shared by these pathways, microRNAs involved in the merged network were overlapped.miR-9, miR-200c, and miR-182 in the Udown group were involved in all the three pathways; in contrast, miR-153was predicted to be exclusively involved in the focal adhesion pathway. More than 40 microRNAs in the Uup group were shared by the above pathways, while miR-503, miR-122, miR-495, and miR-382 were exclusively involved in the focal adhesion pathway, and miR-150, miR-411, miR-146a/b exclusively participated in the MAPK pathway (S4 Table).

**Table 1:** Overlapping genes and their corresponding regulatory microRNAs in the axon guidance, focal adhesion and MAPK signaling pathways.

ID	Axon guidance and focal adhesion pathways	Axon guidance and MAPK signaling pathways	All three pathways
Shared Uup genes	Rock2, Rock1, Rac1, Ptk2, Pak7, Pak6, Pak1, Met, Mapk3, Mapk1, Itgb1, Gsk3b, Fyn	Rac1, Ppp3r1, Pak1, Nras, Mapk3, Mapk1, Kras	Rac1, Pak1, Mapk3, Mapk1
Corresponding Uup microRNAs	miR-96, miR-125a-3p, miR-185, miR-26a, miR-101a, miR-101b, miR-124, miR-378, miR-16, miR-34a, miR-128, miR-23a, miR-320, miR-138, miR-148a, miR-148b, miR-202-3p, miR-381	miR-96, miR-384-5p, miR-30a, miR-378, miR-16, miR-148b, let-7b, miR-148a, miR-124, let-7f, let-7g, let-7a, let-7d, let-7e, miR-20a, miR-93, miR-106b, miR-17, miR-181a, miR-543, miR-320	miR-16, miR-96, miR-320, miR-378
Shared Udown genes	Rhoa	Rasa1	
Corresponding Udown microRNAs	miR-200c	miR-182	

**Fig 6:**
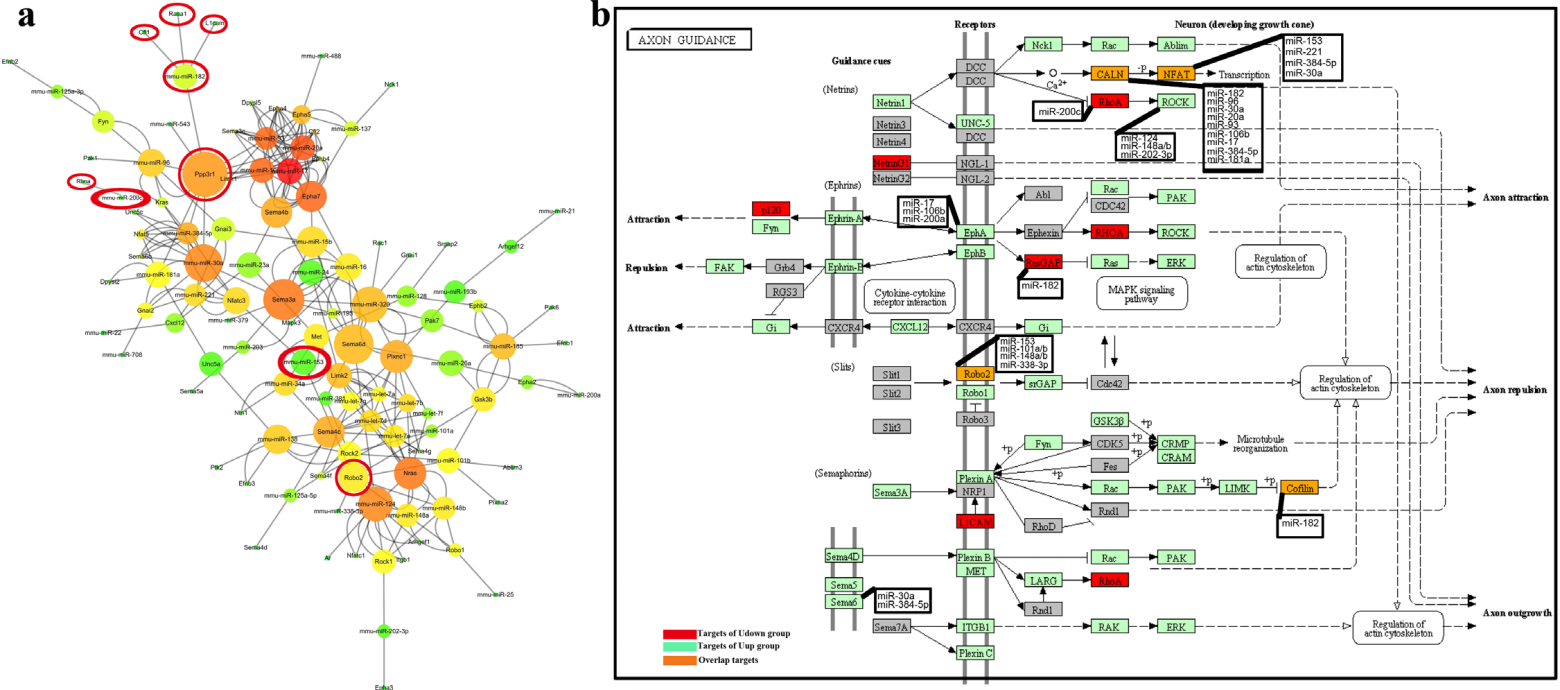
MicroRNA-mRNA interactions in the most reliable and highly-enriched pathway. (a) Axon guidance-related microRNA-mRNA interaction analysis. microRNAs and mRNAs are displayed as nodes. The node size represents the parameters of “weight” and the node color represents the closeness centrality of nodes in the network. The results were analyzed using Cytoscape. Nodes circled with red are microRNAs in the Udown group and their predicted targets. (b) Axon guidance pathway analysis. Green, microRNAs in the Uup group and their targets; red, microRNAs in the Udown group and their targets; orange, genes targeted by both groups of microRNAs.

To further explore the interactions between the two groups of microRNAs, the most highly-enriched pathway in both categories, the axon guidance pathway, was analyzed in detail. The regulatory network showed that 51 differentially-expressed microRNAs and 66 target genes were included in the axon guidance pathway. Among the microRNAs, miR-182 in the Udown group together with 8 microRNAs in the Uup group (miR-96, miR-30a, miR-20a, miR-93, miR-384-5p, miR-106b, miR-17, and miR-181a) targeted *Ppp3r1*. miR-153 in the Udown group targeted *Nfatc3*, along with miR-221, miR-384-5p, and miR-30a in the Uup group. Also, *Robo2* was targeted by miR-153 in the Udown group and miR-148a/b, miR-338-3p, and miR-101a/b in the Uup group (Fig 6A).

The topology and internal interactions of this network were then analyzed with the Network Analyzer plug-in. Node size was scaled by the parameter of betweenness centrality that represents the “weight” of nodes in a network, and the node color reflected the parameter of closeness centrality in the network. The results showed that miR-30a, miR-320, and miR-124 in the Uup group were dominant in node interactions, as reflected by their relatively large size. Three mRNAs *Ppp3r1*, *Sema3a* and *Sema6d* were also dominant in the network in terms of size. Strikingly, miR-17 of the Uup group had close interactions with related nodes, as reflected by the dark red color. Interestingly, miR-182 and miR-153 in the Udown group had close relations with the Uup microRNAs through their targets *Nfatc3*, *Ppp3r1*, and *Robo2* (Fig 6A and Table 1), indicating cooperation between the microRNAs in these two groups in regulating multiple processes.

To explore the specific synergy of these two categories of microRNAs in axon guidance, their targets were further mapped into the axon-guidance map. The results demonstrated that the targets of microRNAs in the Udown group (*Rhoa*, *Caln*, *Nfat*, *NetrinG1*, *P120*, *Robo2*, *L1cam*, and *Cfl1c*o-operated with 57 targets of the microRNAs in the Uup group to regulate the processes of axon attraction, axon repulsion, and axon growth (Fig 6B). Specifically, miR-153in the Udown group microRNA and five microRNAs in the Uup group, miR-148a/b, miR-101a/b, and miR-338-3p, targeted the same mRNA, *Robo2*. Also, miR-153 in the Udown group together with miR-221, miR-384-5p, and miR-30a in the Uup group targeted *Nfat*. miR-182 in the Udown group together with miR-96, miR-30a, miR-20a, miR-93, miR-106b, miR-17, miR-384-5p, and miR-181a in the Uup group targeted *Caln* (Fig 6). It is notable that miR-17, miR-30a, and miR-124 in the Uup group played critical roles in controlling the attraction and repulsion of axons *via* their respective predicted targets. Moreover, miR-182 in the Udown group was potentially involved in axon outgrowth and axon repulsion by acting on the targets *Rasa1*(also named *RasGAP*) and *Cfl1*(also named *Cofilin*) respectively (Fig 6B).

### Validation of Representative microRNAs and mRNAs

Since the network analysis showed that miR-182 in the Udown group and miR-17, miR-30a, and miR-124 in the Uup group play dominant roles in the axon guidance pathway, they and their corresponding target genes were selected for further validation. Meanwhile, miR-200c in the Udown group and miR-200a in the Uup group, as well as their target genes were selected for validation of the accuracy of target prediction, since the interactions between them and their potential target genes are unique.

**Fig 7:**
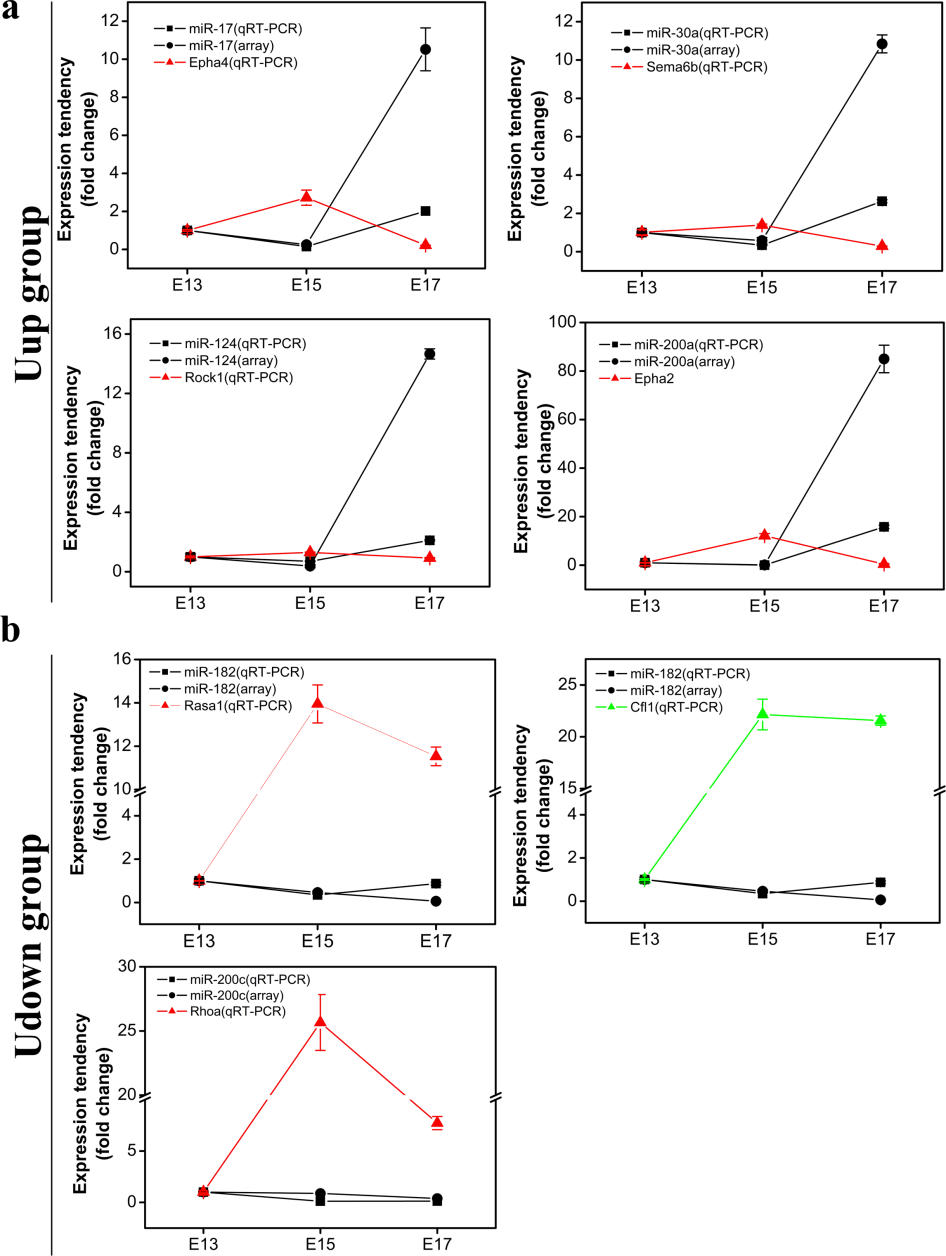
Target prediction validation–quantification of representative microRNAs and mRNAs in the most-enriched pathway. (a) Quantification of representative microRNAs in the Uup group on E13, E15, and E17 by microarray and qRT-PCR. (b) Quantification of representative microRNAs in the Udown group on E13, E15, and E17 by microarray and qRT-PCR. Data are shown as the mean± SD and values were further normalized to that of E13 (n = 3).

The expression of representative microRNAs from the Uup and Udown groups and their potential targets were tested by qRT-PCR at the 3 time-points (Table 2). Quantification showed that the expression patterns of the tested microRNAs in the Uup group were consistent with those of the microRNA arrays except for disparities in the fold changes on E17 (Fig 7A), which were probably due to the differences in data acquiring and normalization between these two techniques. For qRT-PCR, data acquired based on amplification reactions and the fold changes were obtained using 2^-∆∆CT^ algorithm after normalization by the expression of an internal reference. On the other hand, data obtained by microarray based on the hybridization between the microRNAs and the probes, and data of microarray were normalized by median method after background subtraction. If the raw signals from the probes dramatically increased, the calculated fold changes after background subtraction would be larger than the data obtained using 2^-∆∆CT^ algorithm. This is the reason why disparities in fold changes existed between these two techniques when the microRNA levels dramatically increased on E17. It is notable that the expression tendency of the tested genes was negatively correlated with that of their potential regulatory microRNAs. Because the same detection method was used, a tighter negative correlation existed between the expression of mRNAs and that of microRNAs quantitated by qRT-PCR.

**Table 2:** Predicted binding energy between representative microRNAs and mRNAs in the top pathway according to binding dynamics.

Group	Gene	microRNA	ΔΔG^a^	Chromosome^b^
Uup	Epha4	mmu-miR-17	-6.96	1
	Sema6b	mmu-miR-30a	-8.85	17
	Rock1	mmu-miR-124	-9.07	18
	Epha2	mmu-miR-200a	-5.65	4
Udown	Rasa1	mmu-miR-182	-6.64	13
	Cfl1	mmu-miR-182	-7.25	19
	Rhoa	mmu-miR-200c	-6.3	9

The upper sequences are microRNAs and the lower sequences are the complementary 3’UTRs of target genes.

ΔΔG^a^, the binding free energy between microRNA and their potential targets calculated by algorithm of miRanda.

Chromosome^b^, location of microRNAs.

For the tested microRNAs in the Udown group, quantification showed that their expression patterns were generally consistent with those of the microRNA arrays except for a slight deviation on E17, which was probably attributable to the differences in sensitivity between the two methods in detecting the very low expression of microRNAs. It was surprising that a slight reduction in the microRNA level of Udown group could result in dramatic increase in the level of their potential targets, indicating that these microRNAs were their dominant regulators and a threshold, specific level of microRNAs, existed in the inhibitory effects of microRNAs on their respective targets (Fig 7B). It is notable that the expression tendency of tested genes was negatively correlated with that of their potential regulatory microRNAs as assessed by qRT-PCR. Taken together, these data suggested that the fidelity of target prediction was high.

## Discussion

The components of AF are mainly derived from the fetus itself and its composition reflects the dynamic status of the developing fetus. Specific components of AF serve as indicators of fetal developmental and specific physiological conditions [5, 6]. Besides this indictor function, AF has also been used to assess and predict the preterm births [21].

Currently, researchers rely on the high-throughput techniques and bioinformatics to disclose the global and complex biological processes occurring in AF. The transcriptome and the proteome have been extensively used to monitor the status of fetal development by measuring changes in the cell-free mRNAs and proteins in AF [3, 6, 11, 22, 23].It is well-known that AF contains a variety of cell-free components as well as membrane-bound cells, the amniocytes. Among these components, RNAs occur in the form of cell-free particles or bound in amniocytes [24]. The cell-free RNAs originate from amniocytes that secrete or release RNAs under normal conditions or when they undergo programmed cell-death. Some cell-free RNAs may also be derived from fetal tissues that do not directly contact the AF, such as the tongue and intestine, from which secreted RNAs probably diffuse into the AF after fetal swallowing [25, 3]. Among these RNAs, microRNAs, a class of small molecules with critical roles in developmental processes, can be secreted from tissues and packaged in microvesicles, which then deliver these microRNAs to distant recipient tissues to regulate corresponding gene expression [26, 27, 19]. So, to some extent, AF reflects fetal developmental features *per se* while functioning as feedback for fetal development *via* secreted or released microRNAs. However, the functions of AF microRNAs in fetal development have been received little attention. Given that the overall structure and molecular mechanisms of AF development are quite similar in human and rodents, we used the mouse as a model to investigate the functions of AF in fetal development. To investigate their specific functions in fetal development, we profiled the microRNAs of AF at different time-points using microRNA arrays. The array data demonstrated that the global microRNA expression profiles on E13, E15, and E17 constituted two distinct groups, with reversed expression patterns (Fig 1C). Interestingly, the results of gene annotation and pathway mapping of these two groups were consistently enriched in the axon guidance, focal adhesion, and MAPK signaling pathways (Figs 3 and 4). The axon guidance pathway was the most enriched pathway in both groups with the highest fidelity, suggesting synergy of these microRNAs in fetal development. Topological analysis of the microRNA-mRNA networks and axon-guidance pathway mapping showed that the synergy of these microRNAs was accomplished in two ways, one through targeting the same mRNA, and the other through the cooperation of target mRNAs in the same process (Fig 6). Besides the above overlapping pathways of two groups of microRNAs, pathways in cancer were also highly enriched at the different time points. It is known that from E13 to E17, fetal mice undergo rapid growth and proliferation, and the morphology and biological properties of embryonic cells are similar to those of tumor cells. Moreover, stem cells in AF can effectively target tumors and suppresses tumor growth by releasing cytokines, which probably partly explains how the process of fetal development is under strict programming rather than showing disturbed and excessive growth [28, 29].

One of the pronounced features of development is that the embryonic skin is non-keratinized, so small molecules can be easily transferred into the fetus [9]. Skin is innervated by the peripheral processes of somatosensory neurons early in development [30]. Embryological experiments suggest that the skin attracts sensory processes by releasing guidance cues [31]. Moreover, the dynamic changes of peripheral processes require extrinsic cell types [32]. AF microRNAs probably participate in axon guidance as cellular signaling cues. Our data showed that the most highly-enriched pathway of AF microRNAs is axon guidance, suggesting that these microRNAs are crucial in the development of sensory nerves.

## Conclusions

Our work is the first study on the potential functions of AF microRNAs in fetal development. Here, we screened 233 differentially-expressed microRNAs that were divided into two distinct groups. The microRNAs in these two groups work in synergistically in the axon guidance, focal adhesion, and MAPK signaling pathways, which are critical for the normal formation of the nervous system and other organs. The data provide novel insights into the functions of AF in fetal development.

## Supporting Information

S1 Table Reverse-transcription primers of qRT-PCR. (XLSX)

S2 Table Amplification primers of qRT-PCR. (XLSX)

S3 Table Clusters and target prediction of differentially-expressed microRNAs. (XLSX)

S4 Table Overlapping microRNAs in the axon guidance, focal adhesion and MAPK signaling pathways. (XLSX)
